# Microbubble contrast agent SonoVue: An efficient medium for the preoperative lymphatic mapping of thyroid carcinoma

**DOI:** 10.3389/fbioe.2022.1077145

**Published:** 2022-12-08

**Authors:** Lei Chen, Bingwan Dong, Liu Jiang, Jixin Zhang, Luzeng Chen, Tiancheng Li, Yuhong Shao, Xiuming Sun

**Affiliations:** ^1^ Department of Ultrasound, Peking University First Hospital, Beijing, China; ^2^ Department of ORL-HNS, Peking University First Hospital, Beijing, China; ^3^ Department of Pathology, Peking University First Hospital, Beijing, China

**Keywords:** lymphatic contrast-enhanced ultrasound, sentinel lymph node, thyroid carcinoma, metastasis, microbubble contrast agent

## Abstract

**Objective:** To assess the value of microbubble contrast agent SonoVue in the thorough preoperative lymphatic mapping of patients with thyroid carcinoma, including the lymphatic drainage region, the detection of sentinel lymph node (SLN), and the diagnosis of lymph node metastasis (LNM).

**Materials and methods:** 55 patients with 62 thyroid malignancies proved by surgical pathology (59 papillary thyroid carcinomas and three medullary thyroid carcinomas) who underwent preoperative lymphatic contrast-enhanced ultrasound (LCEUS) with microbubble contrast agent SonoVue were enrolled. All LNM were confirmed by pathology. The location of thyroid lesions, ultrasonic features of lymph nodes, lymphatic drainage region, and detection of SLN were assessed. The diagnostic performance (sensitivity, specificity, positive predictive value, negative predictive value and accuracy) of different parameters for the LNM diagnosis was calculated.

**Results:** SonoVue effectively demonstrated the lymphatic drainage region for all enrolled thyroid carcinomas. The most common lymphatic drainage region for thyroid carcinomas was region VI (93.55%), followed by region III (62.90%), region IV (48.39%) and region II (4.84%). When divided by the lesion location, the most common lymphatic drainage regions for the nodule in isthmus, superior lobe and inferior lobe of the thyroid were region VI, region III, and region VI respectively. SLN was detected in 96.77% (60/62) of cases. The two cases without SLN demonstration had pathologically proven LNM. The most common sonographic sign of LNM was perfusion defect (54.17%). The diagnostic accuracy of SonoVue in central and lateral compartment LNM was 86.67% and 91.67%, respectively.

**Conclusion:** Microbubble contrast agent SonoVue is a valuable imaging contrast medium for thorough preoperative lymphatic mapping in patients with thyroid carcinoma, including the lymphatic drainage region, the detection of SLN, and the diagnosis of LNM. LCEUS with SonoVue alone has limitations of false negatives when there is lymphatic vessel obstruction and may need to be combined with other ultrasound modalities.

## 1 Introduction

The incidence of thyroid cancer, which primarily involves papillary thyroid carcinoma (PTC), is increasing gradually worldwide (Cabanillas et al., 2016; Kowalska et al., 2016; Araque et al., 2020). In total, 30%–80% of PTC patients have lymph node metastasis (LNM). Although the incidence of LNM in patients with follicular thyroid carcinoma (FTC) and medullary thyroid carcinoma (MTC) is relatively lower, LNM is still an adverse prognostic factor associated with higher recurrence and mortality rates (Haugen et al., 2015), and affects the staging and surgical strategy for patients with thyroid carcinoma. Therefore, preoperative accurate evaluation of lymphatic drainage routes and potential LNMs for patients with thyroid carcinoma is essential.

However, as the first-line imaging modality for lymph node (LN) disease, conventional ultrasound (US) has limited value in the diagnosis of LNM from thyroid carcinoma, especially in the central compartment (Choi et al., 2009; Hwang and Orloff, 2011; Nie et al., 2017; Garau et al., 2020). Prophylactic and therapeutic dissections of central or lateral neck compartments in addition to thyroidectomy can detect subclinical LNM, playing an important role in the treatment and staging of differentiated thyroid cancer (Carling and Udelsman, 2014; Garau et al., 2020). Yet, systematic LN dissection could destroy the normal lymphatic channels and lead to other related complications namely laryngeal nerve injury and permanent hypoparathyroidism (Chen et al., 2018; Simescu et al., 2019; Garau et al., 2020). Thus, preoperative lymphatic mapping is crucial for surgery planning.

At present, the techniques for lymphatic mapping of thyroid carcinoma mainly include vital blue dye and radioisotope (Garau et al., 2018; Albers et al., 2020; Garau et al., 2020). Their disadvantages, including allergic reactions, ionizing radiation, invasiveness and inconvenient operation, limit their clinical implementation. An alternative lymphatic mapping technique is still in demand.

SonoVue is a second-generation pure blood pool ultrasound contrast agent (Barr et al., 2020). It consists of SF6 microbubble with a phospholipid monolayer shell, with the number mean diameter of 1.9 ± 0.1 µm and a volume median diameter of 8.0 ± 0.9 µm (Frinking et al., 2020), which can be rapidly removed from the blood by the pulmonary route after injection (Schneider, 1999). It has the advantages of safety, easy implementation and a low allergy rate. And it is dominant in the detection of tissue perfusion (Chen et al., 2021). Recently, SonoVue has been applied in the sentinel lymph node (SLN) detection and diagnosis of thyroid cancer. A previous study indicated that lymphatic contrast-enhanced ultrasound (LCEUS) with SonoVue was an effective technique for identifying SLN and diagnosing LNM from PTC (18). However, studies in this area are rare and a thorough preoperative evaluation of lymphatic routes in thyroid carcinoma lacks.

In this study, we aimed to assess the value of SonoVue in the thorough preoperative lymphatic mapping in patients with thyroid carcinoma, including the lymphatic drainage region, the detection of SLN, and the diagnosis of LNM.

## 2 Materials and methods

### 2.1 Patients

This study was approved by the Ethics Committee of Peking University First Hospital. All patients signed informed consent before the examinations.

#### 2.1.1 Inclusion criteria

1. Surgical pathology confirmed thyroid carcinoma; 2. US and LCEUS examination within 3 months before surgery; 3. LNs with pathological results, and were visible on conventional ultrasound.

#### 2.1.2 Exclusion criteria

1. Pediatric or pregnant patients; 2. Without identifiable thyroid lesion; 3. Patients with thyroidectomy or radiotherapy history; 4. Patients with pathology confirmed LNM from other sites.

Finally, from March 2022 to September 2022, 55 patients with 62 thyroid malignancies proved by surgical pathology (59 papillary thyroid carcinomas and three medullary thyroid carcinomas) were enrolled, including 20 males and 35 females, with an average age of 41.16 ± 10.13 years.

### 2.2 Ultrasound examination

All examinations were performed by two physicians with more than 8 years of experience of thyroid ultrasound scanning, using a Mindray Resona R9 (Mindray Global, Shenzhen, China) ultrasonic system equipped with a 4–15 MHz linear transducer according to a standard department protocol, which was consistent during the study period.

The location (isthmus, superior or inferior left/right lobe) of thyroid nodules was recorded. The distribution (central/lateral compartment), size, shape (long-axis diameter/short-axis diameter ratio, L/S ratio), margin (well-defined/ill-defined), echogenicity (hypoechoic, isoechoic or hyperechoic with respect to adjacent muscles), calcification (present/absent), and necrosis (present/absent) of cervical LNs were recorded. LNs were recorded as suspicious/not suspicious for LNM based on previous literature (Chen et al., 2020).

### 2.3 Lymphatic contrast-enhanced ultrasound examination with SonoVue

SonoVue (Bracco, Milan, Italy) was used as an ultrasound contrast agent. A 5.0 ml solution of 0.9% saline and SonoVue was mixed by oscillation. 0.8 ml SonoVue was injected directly into the peritumoral thyroid parenchyma (≤2 mm from the lesion, in front of the tumor was preferred and on the lateral side was an option when there is no safe route) under US guidance. The mechanical index (MI = 0.08–0.09) was selected automatically by the ultrasonic system. Associated complications were recorded.

Three parameters were obtained: The lymphatic drainage region was identified by tracing the enhanced afferent lymphatic vessel within 60 s after injection. The division of cervical LNs (regionⅠ-Ⅶ) was based on the 8th edition of the American Joint Committee on Cancer (AJCC) Manual (Amin et al., 2017). The SLN was identified by tracing the lymphatic vessel and with contrast enhancement, and the LCEUS features of SLN and suspicious LNM on the US were recorded by 300 s of observation after injection of SonoVue. The features of LNs were recorded as follows: (Araque et al., 2020) perfusion: no perfusion, perfusion defect, complete perfusion; (Kowalska et al., 2016) ring sign: complete or incomplete (Wei et al., 2021).

All images were reviewed, blind to the histological information, by two physicians with more than 8 years of experience in a consensus manner.

### 2.4 Statistical analysis

SPSS 16.0 software (IBM, Armonk, NY, United States) was used for statistical analysis. Continuous data with a normal distribution were described by mean ± standard deviation and categorized data were described by percentage. Using independent sample *t*-test for the comparison of continuous data with a normal distribution. The diagnostic performance [sensitivity, specificity, positive predictive value (PPV), negative predictive value (NPV) and accuracy] for LNM based on US and LCEUS with SonoVue were calculated. *p* < 0.05 (two-tailed) was considered to be statistically significant.

## 3 Results

### 3.1 The ultrasound features

The majority of thyroid nodules were located in the inferior left/right lobe (28/62, 45.16%), followed by the superior left/right lobe (26/62, 41.94%) and isthmus (8/62, 12.90%).

Eighty-four LNs were enrolled, including 60 (71.43%) in central compartment and 24 (28.57%) in lateral cervical compartment. Forty-eight (57.14%) LNs were found metastasis of thyroid carcinoma, and no metastasis were identified in the other 36 (42.86%) LNs. The long-axis and short-axis diameters of LNs with metastasis (9.19 ± 5.21 mm, 5.58 ± 2.94 mm) were larger than the benign ones (6.63 ± 2.09 mm, 3.99 ± 1.20 mm) (*p* = 0.003, *p* = 0.001, respectively). The diagnostic performance of US for LNM was 67.74% sensitivity, 41.38% specificity, 55.26% PPV, 54.55% NPV and 55.00% accuracy in central compartment, and 82.35% sensitivity, 71.42% specificity, 87.50% PPV, 62.50% NPV, and 79.17% accuracy in lateral compartment (Chen et al., 2020). As summarized in Table 1.

**TABLE 1:** Diagnostic performance of US and LCEUS with SonoVue for LNM in the central and lateral cervical compartments.

Modalities	LN location	Sensitivity (%)	Specificity (%)	PPV (%)	NPV (%)	Accuracy (%)
US	Central compartment	67.74	41.38	55.26	54.55	55.00
	Lateral compartment	82.35	71.42	87.50	62.50	79.17
LCEUS with SonoVue	Central compartment	87.10	86.21	87.10	86.21	86.67
	Lateral compartment	88.24	100.00	100.00	77.78	91.67

### 3.2 Lymphatic drainage region

Lymphatic drainage was successfully observed in all cases by LCEUS with SonoVue. The most common lymphatic drainage region for thyroid carcinomas was region VI (58/62, 93.55%), followed by region III (39/62, 62.90%), region IV (30/62, 48.39%) and region II (3/62, 4.84%). When divided by the nodule location, the most common lymphatic drainage region for the nodule in the isthmus was region VI (8/8, 100.00%), followed by region IV (4/8, 50.00%); the most common lymphatic drainage region for the nodule in superior lobe was region III (23/26, 88.46%), followed by region VI (22/26, 84.62%); and the most common lymphatic drainage region for the nodule in the inferior lobe was region VI (28/28, 100.00%), followed by region IV (14/28, 50.00%).

Yet, based on pathology results, there were four cases with LNM without lymphatic drainage demonstration to the corresponding area on LCEUS with SonoVue.

### 3.3 Sentinel lymph node detection

**FIGURE 1:**
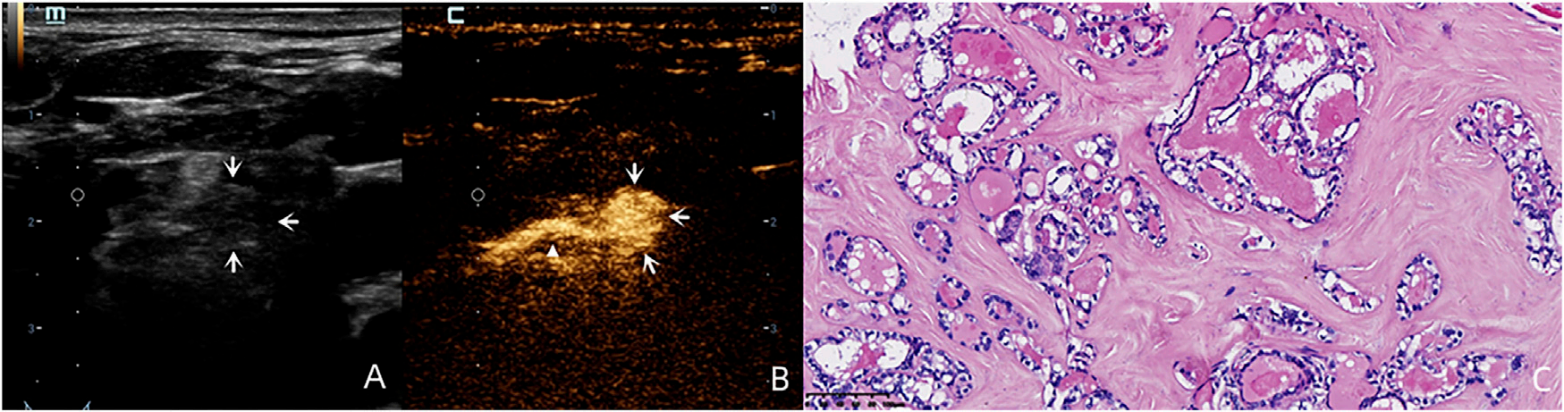
A 59-year-old female patient with pathology proved PTC **(C)** in the left inferior lobe of the thyroid. By following the enhanced drainage lymphatic vessel (white triangle), lymphatic drainage region was observed to region Ⅵ, and the SLN was identified (white arrows). Although this lymph node was round in shape without a clear hilum structure on the US **(A)** and was diagnosed as suspicious for LNM on the US, this lymph node demonstrated complete perfusion after injection of SonoVue **(B)** and was pathology proved benign.

By following the enhanced drainage lymphatic vessel, SLN was detected in 96.77% (60/62) of cases (Figure 1). The two cases without SLN demonstration were found to have pathologically proven LNM.

### 3.4 Lymph node metastasis diagnosis by SonoVue

**FIGURE 2:**
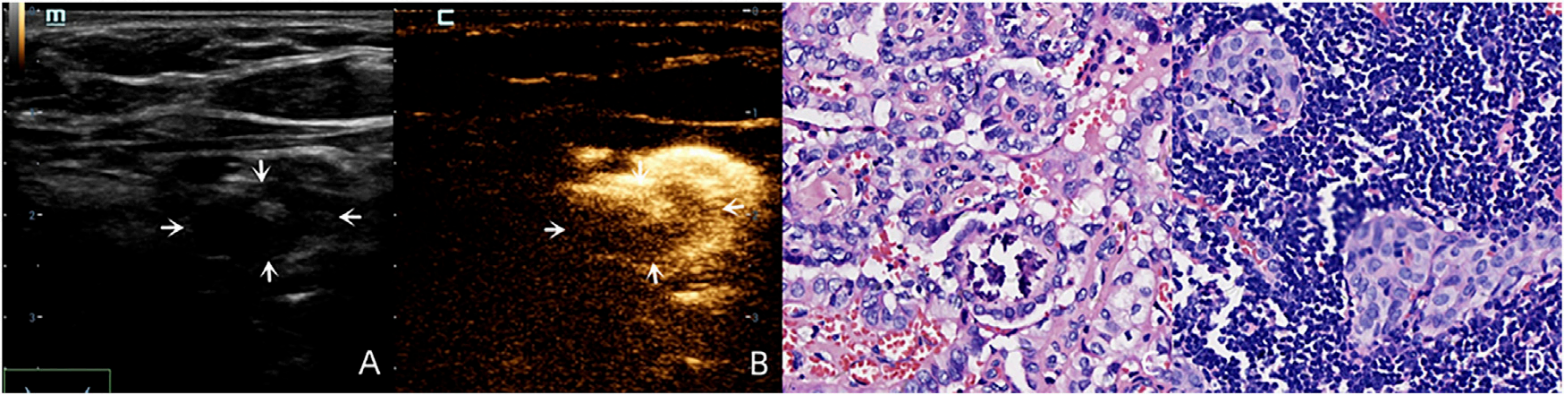
A 30-year-old female patient with pathology proved PTC **(C)** in the left superior lobe of the thyroid. Lymphatic drainage region was observed in regions Ⅲ, Ⅳ and Ⅵ. There was a suspicious metastatic lymph node (white arrows) in region Ⅵ with an oval shape (L/S < 2) and calcification on the US **(A)**, and this lymph node demonstrated a perfusion defect after injection of SonoVue **(B)** and was pathology proved LNM **(D)**.

**FIGURE 3:**
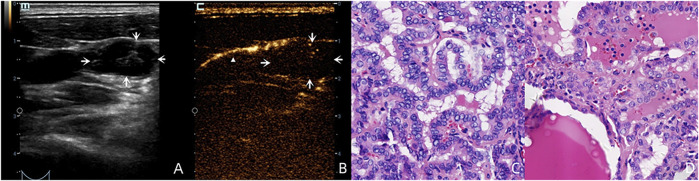
A 36-year-old male patient with pathology proved PTC **(C)** in the left superior lobe of the thyroid. LCEUS with SonoVue demonstrated lymphatic drainage with an enhanced lymphatic vessel (white triangle) to the left region Ⅲ **(B)**. However no SLN was shown because this suspicious lymph node **(A)**, white arrows with round shaped and cystic structure on the US had no perfusion on LCEUS with SonoVue **(B)**, white arrows which made it “invisible.” This lymph node was pathology proved LNM **(D)**.

For the benign lymph nodes, the most common feature after injection of SonoVue was complete perfusion (27/36, 75.00%). For LNM, the most common feature was perfusion defect (26/48, 54.17%), followed by no perfusion (16/48, 33.33%) and interrupted lymphatic vessel (7/48, 14.58%) (Figures 2, 3). If using the presence of perfusion defects or no perfusion as the diagnostic criteria for LNM (Liu et al., 2021; Wei et al., 2021; Wei et al., 2022), the diagnostic performance of SonoVue in central compartment LNM was 87.10% sensitivity, 86.21% specificity, 87.10% PPV, 86.21% NPV, and 86.67% accuracy, respectively. The diagnostic performance of SonoVue in lateral compartment LNM was 88.24% sensitivity, 100.00% specificity, 100.00% PPV, 77.78% NPV, and 91.67% accuracy, respectively (Table 1).

### 3.5 Associated complications

About half of enrolled patients (30/55, 54.55%) reported mild pain at the injection point. No severe complication was reported.

## 4 Discussion

Our results had similar findings as previous reports (Zhao and Li, 2019), and demonstrated that conventional US had limited value in identifying cervical LNM of thyroid carcinoma, especially in the central compartment. Some sonographic features were reportedly associated with the LNM from thyroid carcinoma, such as hyperechogenicity, round shape, absence of hilum structure, calcification, and necrosis (Chen et al., 2021), yet these features may overlap with other lymph node diseases. Lymph nodes in central compartment more frequently show smaller sizes, round shapes with unclear hilum structures, which leads to a false positive or negative diagnosis.

To minimize surgical complications and reduce unnecessary systematic lymph node dissection, continuous efforts have been made on finding new methods for preoperative lymphatic drainage routes and SLN demonstration. As one of the most commonly used contrast agents in ultrasound, SonoVue is mostly administered intravenously to evaluate tissue perfusion. It has low solubility in blood with good stability, helping obtain an optimal backscattered signal with non-linear characteristics. In our study, we found that SonoVue injection into the thyroid parenchyma (LCEUS) had a satisfying performance in drawing lymphatic drainage routes. This demonstrated that SonoVue microbubbles could create a boundary layer with a high impedance mismatch not only in blood but also in tissue and lymphatic channels, resulting in a strong ultrasound signal which could distinguish the lymphatic routes from the background. For overall thyroid carcinoma, the most common lymphatic drainage region was to region VI. Nevertheless, the lymphatic drainage region varied based on different thyroid lesion locations. For thyroid lesions in the superior lobe, the most common lymphatic drainage region was to region III, while for lesions in the isthmus and the inferior lobe, the most common lymphatic drainage region was to region VI, which was consistent with the previous studies (Nie et al., 2017; Garau et al., 2020). We used observation within 60 s after the SonoVue injection in tracing lymphatic drainage to avoid confusion caused by the diffusion of SonoVue in the thyroid parenchyma. This technique is essential since “skip metastases” exist in both the ipsilateral and contralateral compartments (Attard et al., 2019; Garau et al., 2020), and using SonoVue to preoperatively visualize lymphatic drainage routes could benefit the targeted search for LNM and surgery planning. Interestingly, we found that several thyroid carcinoma cases with LNM were without lymphatic drainage shown into the corresponding cervical region. This might be due to the lymphatic vessels blockade by metastatic tumor cells, and might lead to false negative findings.

For the SLN detection, SonoVue also showed an over 90% detection rate. At present, the two commonly used techniques for thyroid carcinoma SLN detection, vital blue dye and radioisotope, have associated limitations and heterogeneity in accuracy (Rubello et al., 2006; Garau et al., 2018; Garau et al., 2020). In this study, we found SonoVue to be an efficient imaging medium for SLN detection in thyroid carcinoma (Liu et al., 2021), with the advantages of low price, real-time operation, low invasiveness and safety. However, similar to the lymphatic drainage region, SLN failed to be detected in two cases where cervical LNM was found. We speculated that this might be due to non-visualization of SLN associated with lymphatic channel disruption by metastasis or accumulation of tumor cells inside the LN. Thus, we state that this technique needs to be combined with other US modalities, such as the conventional US or intravenous CEUS.

**FIGURE 4:**
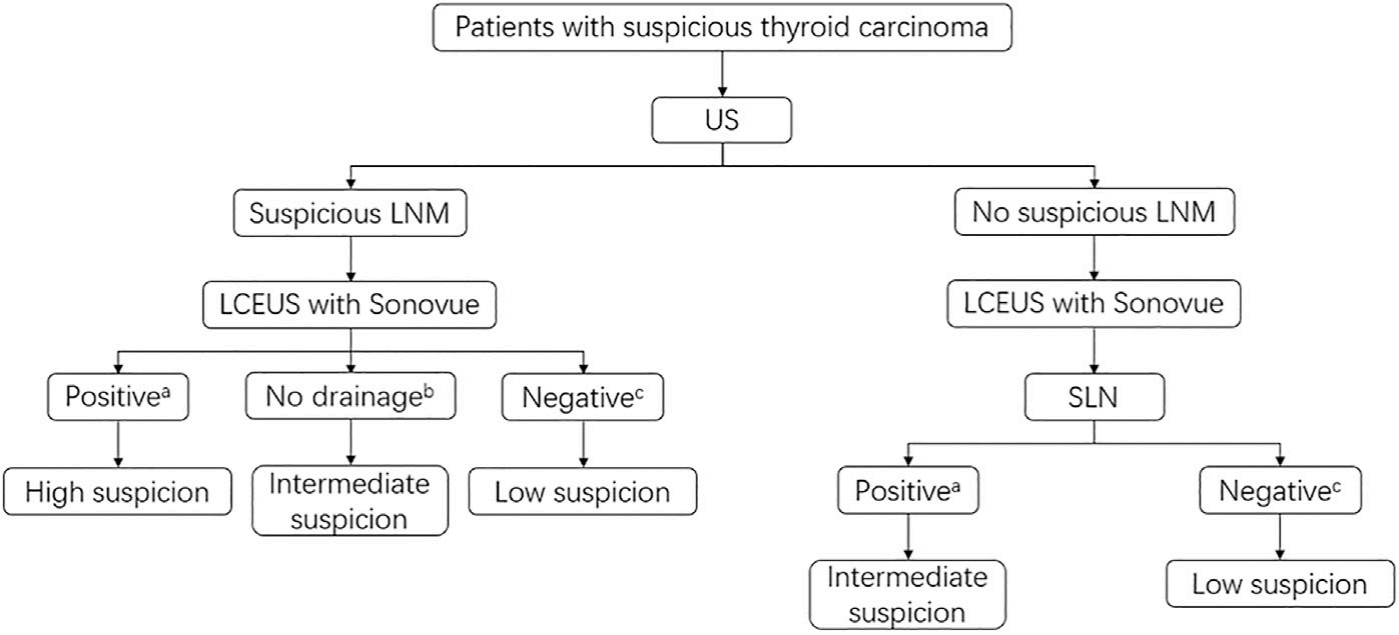
Recommended clinical workflow of LCEUS with SonoVue in the LNM detection and management. ^a^Positive means perfusion defect or no perfusion on LCEUS; ^b^No drainage means no enhanced lymphatic vessels around the suspicious LNM area; ^c^Negative means complete perfusion or complete ring sign on LCEUS. High suspicion: Lymph node biopsy or resection if a biopsy is not operable is recommended; Intermediate suspicion: Further examination such as intravenous CEUS or lymph node biopsy is recommended; Low suspicion: Conventional US follow-up is recommended.

In this study, we also assessed the value of SonoVue in LNM diagnosis. We found that perfusion defects, representing tumor cell assembly in lymph nodes (Wei et al., 2021), were the best feature for indicating LNM. When compared with the conventional US, LCEUS with SonoVue could significantly increase the diagnostic accuracy for LNM in both the central compartment and lateral compartment. Nevertheless, a better performance was found in the lateral compartment. The possible reasons for this phenomenon might be as follows: (Araque et al., 2020). Lymph nodes in the central compartment were usually smaller, making observation for LCEUS features more difficult; (Kowalska et al., 2016). The anatomy of the central compartment was more complicated. Structures such as parathyroid glands and cross-sections of blood vessels might be misdiagnosed as lymph nodes; (Cabanillas et al., 2016). Some lymph nodes in the central compartment might be located behind the thyroid. When administrating SonoVue, the acoustic attenuation caused by diffused contrast agent in the thyroid parenchyma made these lymph nodes invisible. To generalize LCEUS with SonoVue in LNM diagnosis, we made a recommendation for the workflow in clinical settings (Figure 4).

The present study evaluated a microbubble contrast agent, SonoVue, for the thorough preoperative lymphatic mapping in patients with thyroid carcinoma, which might potentially benefit the clinical management of these patients and decline associated surgical complications. This technique evaluates three aspects: lymphatic drainage region, SLN detection and LNM diagnosis. The previous studies mainly focused on the imaging features of LNM (Liu et al., 2021; Wei et al., 2021; Wei et al., 2022). This study emphasized thorough preoperative lymphatic mapping, aiming to provide more comprehensive information for clinical settings. The techniques to perform LCEUS with SonoVue was found to be safe in our study, with mild pain at the injection points and no other severe complications reported.

At present, there are two commonly used ultrasound contrast agents in clinic, namely SonoVue an Sonazoid (Barr et al., 2020). Sonazoid consists of perfluorobutane (C4F10) microspheres stabilized by a monomolecular membrane of hydrogenated egg yolk phosphatidyl serine. It has the number mean diameter of 2.1 ± 0.1 µm and volume median diameter of 2.6 ± 0.1 µm after reconstitution (Frinking et al., 2020). Compared with SonoVue, Sonazoid microbubbles are more stable in the body and enable scanning up to 10–60 min after injection, which is not suitable in our study due to the requirement for repeated injection of contrast agent (resulting in excessive examination time).

There are some limitations of this study: 1. This is a retrospective study. To minimize bias, two experienced physicians blinded to the pathology results reviewed the images. 2. The sample size is relatively small. A prospective larger cohort study will be helpful to generalize the results of this study. 3. The technique of intravenous CEUS was not evaluated in this study, which will be further discussed in our future study.

## 5 Conclusion

SonoVue is a valuable contrast imaging medium for thorough preoperative lymphatic mapping in patients with thyroid carcinoma. Lesions in different thyroid locations have different lymphatic drainage regions. SonoVue could efficiently identify the SLN of thyroid carcinoma and increase the diagnostic accuracy of LNM compared with the conventional US. The most valuable sign for LNM is perfusion defect. However, SonoVue has limitations of false negatives when there is lymphatic channel obstruction and less accuracy in the diagnosis of central compartment LNM compared to the lateral compartment. In clinical practice this technique may need to be combined with other ultrasound modalities.

## Data Availability

The data that support the findings of this study are available from the corresponding authors upon request.
